# Association between STAT4 Gene Polymorphisms and Autoimmune Thyroid Diseases in a Chinese Population

**DOI:** 10.3390/ijms150712280

**Published:** 2014-07-11

**Authors:** Ni Yan, Shuai Meng, Jiaozhen Zhou, Jian Xu, Fatuma Said Muhali, Wenjuan Jiang, Liangfeng Shi, Xiaohong Shi, Jinan Zhang

**Affiliations:** Department of Endocrinology, Jinshan Hospital of Fudan University, No. 1508 Longhang Road, Jinshan District, Shanghai 201508, China; E-Mails: bapintanhua@163.com (N.Y.); momingfd@163.com (S.M.); zhoujiaozhen@163.com (J.Z.); wendy20140710@163.com (J.X.); muhalifatma@yahoo.co.uk (F.S.M.); hidyfan@yeah.net (W.J.); slf8181@163.com (L.S.)

**Keywords:** STAT4, Graves’ disease, Hashimoto’s thyroiditis, single nucleotide polymorphism (SNP)

## Abstract

The STAT4 gene encodes a transcriptional factor that transmits signals induced by several key cytokines which play important roles in the development of autoimmune diseases. The aim of this study was to explore the association of STAT4 polymorphism with Graves’ disease (GD) and Hashimoto’s thyroiditis (HT). A total of 1048 autoimmune thyroid diseases (AITDs) patients (693 with GD and 355 with HT) and 909 age- and gender-matched controls were examined. STAT4 polymorphisms (rs7574865/rs10181656/rs7572482) were genotyped by multiplex polymerase chain reaction (PCR) and ligase detection reaction (LDR). The results indicated that the frequencies of rs7574865 genotypes in patients with GD differed significantly from the controls (*p* = 0.028), the T allele frequency of GD patients was also significantly higher than the controls (*p* = 0.020). The genotypes of rs10181656 differed significantly in GD patients from controls (*p* = 0.012); G allele frequencies were significantly higher in AITD patients than the controls (*p* = 0.014 and 0.031, respectively). The frequencies of haplotype GC with GD and HT patients were significantly lower than their controls (*p* = 0.015 and 0.030, respectively). In contrast, the frequencies of haplotype TG with GD and HT patients were significantly higher than their controls (*p* = 0.016 and 0.048, respectively). These findings strongly suggest that STAT4 rs7574865/rs10181656 polymorphisms increase the risk of AITD in a Chinese population.

## 1. Introduction

Autoimmune thyroid diseases (AITDs), mainly including Graves’ disease (GD) and Hashimoto’s thyroiditis (HT), are organ-specific autoimmune disorders. The precise etiology of AITD is not completely understood but accumulating data on genetic risk factors have significantly advanced our understanding of its pathogenesis. During the past decade, substantial progress has been made in identifying susceptibility genes for AITD involved in immunoregulatory genes like HLA Class II gene [1,2,3], cytotoxic T lymphocyte-associated factor 4 (CTLA-4) gene [4,5,6,7], CD40 gene [4,7,8], protein tyrosine phosphatase-22 (PTPN22) gene [4], and others thyroid-specific genes for the thyroid stimulating hormone (TSH) receptor (TSHR) [4,9,10,11] and thyroglobulin (TG) [12,13], as well as the neotype proinflammatory cytokine such as IL-17 [14], IL-21 [15].

The signal transducer and activator of transcription 4 (STAT4) gene, located on human chromosome 2q32.3, consists of 24 exons spanning a 120 kb region. STAT4 belonging to the STAT family expressed in lymphocytes, macrophages, and dendritic cells, encodes a transcription factor that resides in the cytosol. It transmits the intracellular signals induced by cytokines including interleukin-12 (IL-12), IL-23, IL-27 and type-1 interferons (IFNs) [16]. Upon cytokine signaling, this transcription factor becomes phosphorylated and translocates to the nucleus to play an essential downstream role in the differentiation and proliferation of IL-12-dependent T helper 1 (Th1) cells [17]. STAT4 is also crucial in the development of Th17 in response to IL-23 [18]. Since Th1 cells and Th17 cells are broadly implicated in chronic inflammatory and autoimmune diseases, STAT4 may play an important role in the pathogenesis of AITD.

Investigating the genetic polymorphism of STAT4 with autoimmune diseases, many recent studies suggested a significant association of STAT4 gene polymorphism with systemic lupus erythematosus (SLE) [19,20,21,22], rheumatoid arthritis (RA) [23,24,25,26], Crohn’s disease [27,28], Sjögren’s disease (SD) [29], systemic sclerosis [30,31], Behcet’s disease [32,33], and type-1 diabetes (T1D) [34,35,36] as well as psoriasis [37], thus indicating common genetic involvement in multiple autoimmune diseases. Until now, there are only limited data including a study reporting a significant association of the STAT4 gene single nucleotide polymorphism (SNP) (rs11889341 and rs7574865) with AITD in the Korean population [34]. However, studies of a genetic relationship between STAT4 polymorphisms and AITD in Chinese population have not been conducted so far. In this study, we therefore carried out a large case-control study in a cohort of the Chinese Han population to investigate the genetic association between the selected SNP of STAT4 (rs7574865/rs10181656/rs7572482) and AITD. Furthermore, we analyzed the association between genotypes and AITD clinical characteristics.

## 2. Results and Discussion

### 2.1. Association of STAT4 Polymorphism with AITD (Autoimmune Thyroid Disease)

Genotype distributions for SNP rs7574685, rs10181656, rs7572482 were in accordance with the predicted Hardy-Weinberg equilibrium in both patient and control groups (Table 1). All the allele and genotypic frequencies of these three SNPs in AITD patients and healthy controls are presented in Table 2 and Table 3. Two SNPs (rs7574865 and rs10181656) were significantly associated with AITD (*p* < 0.05) in the Chinese Han population.

**Table 1:** Hardy-Weinberg *p* value of the three SNPs (single nucleotide polymorphisms) in the STAT4 gene. GD, Graves’ disease; HT, Hashimoto’s thyroiditis.

SNP ID	Control	GD	HT
rs7574865	0.6007	0.2293	0.5841
rs10181656	0.3984	0.185	0.8463
rs7573482	0.978	0.6558	0.8889

**Table 2:** Analysis of genotype and allele distribution of three STAT4 SNPs in AITD (autoimmune thyroid disease) patients and controls. OR: odds ratio; CI: confidence interval.

SNP	Alleles/Genotypes	AITD	Control	*p*	OR (95% CI)
rs7574865	GG	427 (40.9)	408 (45.2)	0.025	-
	GT	474 (45.4)	404 (44.7)		
	TT	143 (13.7)	91 (10.1)		
	T	760 (36.4)	586 (32.4)	0.010	1.191
	G	1328 (63.6)	1220 (67.6)		1.043–1.361
rs10181656	CC	424 (40.7)	405 (44.9)	0.009	-
	GC	471 (45.2)	408 (45.2)		
	GG	148 (14.1)	89 (9.9)		
	G	767 (36.8)	586 (32.5)	0.005	1.209
	C	1319 (63.2)	1220 (67.6)		1.058–1.380
rs7572482	GG	229 (22.0)	201 (22.3)	0.985	-
	AG	524 (50.3)	451 (49.9)		
	AA	289 (27.7)	251 (27.8)		
	G	982 (47.1)	853 (47.2)	0.945	0.996
	A	1102 (52.9)	953 (52.8)		0.878–1.130

**Table 3:** Analysis of genotype and allele distribution of three STAT4 SNPs in GD, HT patients and controls.

SNP	Alleles/Genotypes	GD (%)	HT (%)	Control (%)	*p*		OR (95% CI)	
		a	b	c	a *vs.* c	b *vs.* c	a *vs.* c	b *vs.* c
rs7574865	GG	287 (41.6)	140 (39.5)	408 (45.2)	0.028	0.152	-	-
	GT	304 (44.1)	170 (48.0)	404 (44.7)				
	TT	99 (14.3)	44 (12.5)	91 (10.1)				
	T	502 (36.4)	258 (36.4)	586 (32.4)	0.020	0.057	1.190	0.838
	G	878 (63.6)	450 (63.6)	1220 (67.6)			1.027–1.379	0.698–1.005
rs10181656	CC	285 (41.4)	139 (39.3)	405 (44.9)	0.012	0.088	-	-
	GC	303 (44.0)	168 (47.5)	408 (45.2)				
	GG	101 (14.6)	47 (13.2)	89 (9.9)				
	G	505 (36.6)	262 (37.0)	586 (32.5)	0.014	0.031	1.202	1.221
	C	873 (63.4)	446 (63.0)	1218 (67.5)			1.038–1.393	1.018–1.464
rs7572482	GG	154 (22.4)	75 (21.2)	201 (22.3)	0.862	0.739	-	-
	AG	351 (51.0)	173 (48.9)	451 (49.9)				
	AA	183 (26.6)	106 (29.9)	251 (27.8)				
	G	659 (47.9)	323 (45.6)	853 (47.2)	0.711	0.467	1.027	0.937
	A	717 (52.1)	385 (54.4)	953 (52.8)			0.892–1.182	0.787–1.116

The frequencies of rs7574865 genotypes in patients with GD (GG, 41.6%; GT, 44.1% and TT, 14.3%) differed significantly from those in the controls (GG, 45.2%; GT, 44.7% and TT, 10.1%) (*p* = 0.028). The frequency of the minor T allele in GD patients was significantly higher than healthy controls (36.4% *vs.* 32.4%; *p* = 0.020, OR = 1.19, 95% CI = 1.027–1.379); however, the genotype and the minor T allele distribution of rs7574865 did not reveal any significant association with HT. Similar results of rs10181656 were found in GD, the genotypes (CC, 41.4%; GC, 44.0% and GG, 14.6%) differed significantly from those in the controls (CC, 44.9%; GC, 45.2% and GG, 9.9%) (*p* = 0.012). Further analysis indicated that the G allele frequencies were significantly higher in GD patient groups than the control groups (in GD, 36.6% *vs.* 32.5%, *p* = 0.014, OR = 1.202, 95% CI = 1.038–1.393; in HT, 37.0% *vs.* 32.5%, *p* = 0.031, OR = 1.221, 95% CI = 1.018–1.464). For the rs7572482 SNP investigated, we could not demonstrate significant differences in the genotype or allele frequencies of AITD patients when compared with those of healthy controls.

After gender stratification (shown in Table 4), the frequencies of rs7574865 genotypes in female GD patient differed significantly from those in the controls (*p* = 0.033), the frequency of the minor T allele in female GD patients was significantly higher than healthy controls (*p* = 0.042, OR = 1.206, 95% CI = 1.007–1.444); the frequencies of rs10181656 genotypes in female GD patient differed significantly from those in the controls (*p* = 0.019), however, the minor allele distribution did not show any significant associations.

**Table 4:** Analysis of genotype and allele distribution of three STAT4 SNPs in GD, HT patients and controls after gender stratification. * Male; ^#^ Female. The red font stands for the genotype and allele distribution of three STAT4 SNPs in female GD, HT patients and female controls.

SNP	Alleles/Genotypes	GD (a)		HT (b)		Control (c)		*p*		OR (95% CI)	
		Male	Female	Male	Female	Male	Female	a *vs.* c		b *vs.* c	a *vs.* c	b *vs.* c
rs7574865	GG	87 (41.0)	200 (41.8)	14 (31.1)	126 (40.8))	141 (45.0)	267 (45.3)	0.488 *	0.033 ^#^	0.199 *, 0.232 ^#^	-	-
	GT	91 (42.9)	213 (44.6)	23 (51.1)	147 (47.6)	132 (42.2)	272 (46.1)					
	TT	34 (16.1)	65 (15.6)	8 (17.8)	36 (11.6)	40 (12.8)	51 (8.6)					
	T	159 (37.5)	343 (35.9)	39 (43.3)	219 (35.4)	212 (33.9)	374 (31.7)	0.227 *	0.042 ^#^	0.078 *, 0.109 ^#^	-	-
	G	265 (62.5)	613 (64.1)	51 (56.7)	399 (64.6)	414 (66.1)	806 (68.3)				1.206 (1.007–1.444) ^#^	-
rs10181656	CC	87 (41.0)	198 (41.5)	14 (31.1)	125 (40.1)	139 (44.4)	266 (45.2)	0.419 *	0.019 ^#^	0.210 *, 0.121 ^#^	-	-
	GC	91 (42.9)	212 (44.4)	23 (51.1)	145 (46.9)	136 (43.5)	272 (46.2)					
	GG	34 (16.1)	67 (14.1)	8 (17.8)	39 (13.0)	38 (12.1)	51 (8.6)					
	G	159 (37.5)	332(35.3)	39 (43.3)	223 (36.1)	212 (33.9)	374 (31.7)	0.227 *	0.083 ^#^	0.078 *, 0.064 ^#^	-	-
	C	265 (62.5)	608 (64.7)	51 (56.7)	395 (63.9)	414 (66.1)	804 (68.3)					
rs7572482	GG	47 (22.3)	107 (22.4)	8 (17.8)	67 (21.7)	74 (23.8)	127 (21.5)	0.909 *	0.776 ^#^	0.634 *, 0.963 ^#^	-	-
	AG	112 (53.1)	239 (50.1)	24 (53.3)	149 (48.2)	160 (51.4)	291 (49.2)					
	AA	52 (24.6)	131 (27.5)	13 (28.9)	93 (30.1)	77 (24.8)	174 (29.3)					
	G	206 (48.8)	453 (47.5)	40 (44.4)	283 (45.8)	308 (49.5)	545 (46.0)	0.824 *	0.503 ^#^	0.368 *, 0.923 ^#^	-	-
	A	216 (51.2)	501 (52.5)	50 (55.6)	335 (54.2)	314 (50.5)	639 (54.0)					

As shown in Table 5, a strong linkage disequilibrium was observed in SNPs rs7574865 and rs10181656, in AITD patients and controls using the Haploview 4.2 (Broad Institute, Cambridge, MA, USA). The frequencies of STAT4 haplotypes in patients with AITD and controls are presented in Table 6. After analyzing the haplotype of those 3 SNPs, we found 2 common haplotypes, which were GC, TG. The frequencies of haplotype GC with GD and HT patients were significantly lower than their control groups, the GC haplotype showed protective influence for AITD (in GD, *p* = 0.015, OR = 0.833, 95% CI = 0.719–0.965; in HT, *p* = 0.030, OR = 0.818, 95% CI = 0.682–0.981). In contrast, the frequencies of haplotype TG with GD and HT patients were significantly higher than their control groups (in GD, *p* = 0.016, OR = 1.199, 95% CI = 1.034–1.389; in HT, *p* = 0.048, OR = 1.202, 95% CI = 1.002–1.442), and conferred significant degree of risk of AITD for both.

**Table 5:** Linkage disequilibrium in AITD patients and controls.

L1	L2	D’		*r^2^*	
		Control	AITD	Control	AITD
rs7574865	rs10181656	0.995	1.000	0.987	0.986
rs7574865	rs7572482	0.057	0.014	0.001	0
rs10181656	rs7572482	0.067	0.021	0.002	0

**Table 6:** Haplotypic association analysis of rs7574865, rs10181656, rs7572482.

Haplotype	GD (a) (Frequency)	HT (b) (Frequency)	Control (c) (Frequency)	*p*		OR (95% CI)	
				a *vs.* c	b *vs.* c	a *vs.* c	b *vs.* c
GC	875 (63.4%)	446 (63.0%)	1217 (67.5%)	0.015	0.030	0.833 (0.719–0.965)	0.818 (0.682–0.981)
TG	502 (36.4%)	258 (36.4%)	582 (32.3%)	0.016	0.048	1.199 (1.034–1.389)	1.202 (1.002–1.442)

### 2.2. Genotype and Clinical Phenotype Correlations

Within GD patients, when we compared ophthalmopathy patients with non-ophthalmopathy ones, no association was found (*p* > 0.05) (Table 7). After adjusting by gender, no relationships were found between ophthalmopathy phenotypes and these SNPs (data not shown).

**Table 7:** The allele and genotype frequencies of three SNP in ophthalmopathy, non-ophthalmopathy GD patients and in subgroups.

SNP		GD				*p*
		Ophthalmopathy		Non-Ophthalmopathy		
*rs7574865*							
GG		52 (40.9)		235 (41.7)		0.972
GT		56 (44.1)		248 (44.0)		
TT		19 (15.0)		80 (14.3)		
G		160 (14.4)		718 (63.8)		0.817
T		949 (85.6)		408 (36.2)		
*rs10181656*							
CC		51 (40.2)		234 (41.6)		0.954
CG		57 (44.9)		246 (43.8)		
GG		19 (14.9)		82 (14.6)		
C		159 (62.6)		714 (63.5)		0.782
G		95 (37.4)		410 (36.5)		
*rs7572482*							
GG		26 (20.5)		128 (22.8)		0.472	
AG		71 (55.9)		280 (49.9)			
AA		30 (23.6)		153 (27.3)			
G		123 (48.4)		536 (47.8)		0.851
A		131 (51.6)		586 (52.2)		

### 2.3. Discussion

STAT4 has been proven to play a crucial role in immune and autoimmune responses. The STAT4 gene encodes a transcription factor that transmits signals induced by type 1 cytokines type1-IFN, IL-12, and IL-23 [16,18]. It plays a key role in the IL-12-induced differentiation of T cells into the Th1 pathway and is involved in the production of IL-17 by Th17 cells, in response to IL-23. The current human evidence that STAT4 has been shown to be involved in Th1- or Th17-mediated diseases, and to be associated with a number of autoimmune diseases [19,20,21,22,23,24,25,26,27,28,29,30,31,32,33,34,35,36,37], indicates that multiple autoimmune diseases share common susceptibility genes, providing a reason to investigate its association with AITD. In this study, we present the first detailed genotype-phenotype analysis of STAT4 gene variants in a large Chinese Han AITD cohort. Our results show that the minor allele T of rs7574865 was significantly associated with an increased risk of AITD. Additionally, the G allele of rs10181656 has conferred positive association with both GD and HT.

The SNP rs7574865, located in the third intron of STAT4, is confirmed as a promising disease-related gene suggestive of increased risk of RA [23,24,25,26], SLE [19,20,21,22], T1D (type 1 diabetes) [34,35,36], Crohn’s disease [28] and Behcet’s disease [32,33] in different ethnic groups, including Europeans [28], American Whites [20], Koreans [34], Japanese [19], Iranian population [21], and especially in the Chinese population [22,23,25,32,36], suggesting rs7574865 polymorphism may contribute to autoimmune disorders. Researchers indicated TT genotype of rs7574865 may be a susceptible factor for Vogt-Koyanagi-Harada (VKH) syndrome in a Chinese Han population, and that GG genotype of this SNP may confer susceptibility in male Behcet’s disease patients [32]. A study on 428 Korean AITD patients and 1060 healthy controls found that the TT genotype of rs7574865 was higher in AITD patients as a whole (OR = 1.5, 95% CI = 1.07–2.1, *p* = 0.009) and in HT (OR = 1.58, 95% CI = 1.01–2.48, *p* = 0.04), which evidently indicated that the TT genotype could increase susceptibility to HT; nevertheless, there was no association between rs7574865 and GD [34]. Another study carried out in the Tunisian population with 159 AITDs patients and 200 healthy controls using TaqMan allelic discrimination assay did not detect any significant associations between rs7574865 polymorphism and AITD [26]. To our knowledge, there is no association study of rs7574865 with AITD in the Chinese population. In contrast, our present study showed no significant difference of TT genotype frequency between HT and healthy controls in the Chinese population (*p* > 0.05), but we found a strong association of TT genotype with GD (TT *vs.* GT + GG; OR (95% CI) = 1.495 (1.103–2.025), *p* = 0.009); in addition, a positive correlation of T allele with the risk of GD (OR = 1.19, *p* = 0.020), is consistent with that observed in RA and SLE in Japanese, and Chinese individuals [19,20,22,23,25], as well as Europeans with Crohn’s disease [28]. This discrepancy may be due to genetic heterogeneity, different sample sizes, different race/ethnic groups and so on.

Rs10181656 SNP showed no association with AITD patients in the Korean population [34]. In agreement with this study, Glas *et al.* [28] did not reveal any significant association with crohn disease (CD) or ulcerative colitis (UC) susceptibility. But the rs10181656 SNP was significantly associated with anticitrullinated protein antibody (ACPA)-positive RA in a Swedish study [24]. In our present study, we found that the G allele of rs10181656 was positively associated with AITD. This result is consistent with reports in Chinese Han RA patients that the frequency of G allele in RA patients was significantly higher than controls (*p* = 0.004, OR = 1.472, 95% CI = 1.132–1.915) [25].

Because analysis of haplotype provides the genetic information of multiple SNPs, this is a powerful method for the identification of genes contributing to complex diseases. In our study, the associated haplotype is located in the third intron of the STAT4 gene. The rate of GC haplotype was lower in both GD and HT, suggesting a protective effect for AITD, while the rate of the haplotype TG was higher, showing a significant risk factor for AITD. This consistency with allele analysis deeply confirms that T allele of rs7574865 was a predisposing factor for AITD.

It is reported that STAT4 has two alternatively spliced isoforms, STAT4α and STAT4β. STAT4β which lacks 44 amino acids at the *C* terminus of the full-length STAT4α is not as efficient as STAT4α in directly inducing IFN-γ gene expression activated by IL-12 in Th1cells [38]. The publication of Abelson *et al.* [39] observed the risk alleles of STAT4 rs7574865 was correlated with higher STAT4 expression level in Peripheral Blood Mononuclear Cell (PBMC) of SLE patients; it also reported that the presence of the rs7974865 polymorphism significantly increases the predictive ability for SLE, within STAT4, the SNP rs7574865 is the strongest predictor for SLE and act additively to increase the risk for SLE [39]. Since the susceptibility SNPs are located within the third intron of the STAT4 gene, and probably has an influence on the level of STAT4 transcription and splice variation, it might also be possible that the putative functional variant could be responsible for a biologic effect on intragenic RNA or other factors; additionally, it is reasonable to speculate that a variant on STAT4 could also affect disease activity in autoimmune diseases through dysregulation of the Th1 and Th17 pathways. However, the precise functional roles of these risk SNPs remain to be elucidated and further studies are needed to clarify this issue.

## 3. Experimental Section

### 3.1. Patients and Controls

The study population (*n* = 1957) consisted of 1048 Chinese AITD patients including 693 patients with GD, 355 patients with HT, and 909 healthy unrelated controls. The subjects were all Chinese Han ethnicity. Patients with AITD were recruited from the outpatient and inpatient settings of the department of Endocrinology, Jinshan Hospital of Fudan University in China. All the healthy controls were recruited from the Health Care Center at the same hospital. All the controls had neither family history of thyroid disease nor any other autoimmune diseases. This study was approved by the Institutional Review Board of Jinshan Hospital of Fudan University, and all subjects provided written informed consent. The demographic characteristics of the AITD study population are summarized in Table 8.

**Table 8:** Demographic characteristics of the AITD patients and controls.

Item	GD	HT	Control
*n*	693	355	909
*Gender*			
Male (%)	213 (30.7)	45 (12.7)	314 (34.5)
Female (%)	480 (69.3)	310 (87.3)	595 (65.5)
*Age*			
Mean ± SD	36.88 ± 14.555	35.01 ± 13.808	38.75 ± 9.144
Range	5–77	4–78	11–72
Onset of age	33.86 ± 14.357	32.63 ± 13.441	-
*Thyroid size*			
Normal size (%)	23 (3.3)	13 (3.7)	-
I degree (%)	130 (18.8)	61 (17.2)	-
II degree (%)	427 (61.6)	245 (69.0)	-
III degree (%)	113 (16.3)	36 (10.1)	-
*Family history*			
Positive (%)	521 (75.2)	276 (77.7)	-
Negative (%)	172 (24.8)	79 (24.3)	-
*Ophthalmopathy*			
With (%)	127 (18.3)	6 (1.7)	-
Without (%)	566 (81.7)	349 (98.3)	-

GD was diagnosed based on clinical and laboratory biochemical evidence of hyperthyroidism by the presence of diffuse goiter, and supported by the positive anti-thyroid stimulating hormone receptor antibody (TRAb) and/or anti-thyroid peroxidase antibody (TPO-Ab) and/or anti-thyroglobulin (Tg-Ab) and/or exophthalmos. HT was diagnosed based on the presence of an enlarged thyroid and either TPO-Ab or Tg-Ab, with or without documented clinical and biochemical hypothyroidism.

### 3.2. Genotyping

From all study participants, peripheral venous blood of 2 mL was collected and genomic DNA was extracted from peripheral blood cells using the Nucleon Bacc kit (TianGen Biotech Co., Ltd., Beijing, China), according to the manufactures’ guidelines. Genotyping of SNPs (rs7574865, rs10181656, rs7572482) was performed by the Shanghai Biowing Applied Biotechnology Company [40] using ligase detection reactions (LDR). This is a well-established platform used by many researchers for studies including SNP genotyping. The target DNA sequences were amplified using multiplex polymerase chain reaction (PCR) with specific primer sequences as follows:

rs7574865 forward: 5'-AGTATGAAAAGTTGGTGAC-3', reverse:5'-AATCCCCTGAAATTCCACTG-3'; rs10181656 forward: 5'-AACTAGCTGGAATCCAACTC-3', reverse: 5'-ACGAGGAAGATGGTGACAAG-3'; rs7572482 forward: 5'-CAGACAGGCACTGAGTTTTC-3', reverse: 5'-CTTTGCTTTCACTCTCTAGG-3'.

After the amplification, we performed a second-round PCR; the reaction mixture consisted of the following components: 1 μL genome DNA (50 ng/μL), 2 μL Buffer (1×), 0.6 μL Mg^2+^ (3 mM), 2 μL dNTP (2 mM), 0.2 μL Taq DNA polymerase (Qiagen, Shanghai, China), 9.8 μL H_2_O, 4 μL Q-solution (1×) and 2 μL Primer mix. The amplification procedure consisted of initial denaturation at 95 °C for 15 min, 35 cycles of denaturation at 94 °C for 30 s, annealing at 56 °C for 60 s and extension at 72 °C for 60 s, followed by a final extension at 72 °C for 7 min (Perkin-Elmer Gene Amp PCR Systems 9600 (Applied Biosystems, Shanghai, China) was used). The ligation reaction was carried out in a final volume of 10 μL containing 1 μL Buffer (1×), 1 μL Prob mix (0.05 pmol/μL), 1 μL of Multi-PCR product (100 ng/μL), 6.95 μL H_2_O, 0.05 μL of 2 U/μL Taq DNA ligase (New England Biolabs, Ipswich, MA, USA). The LDR was performed using 35 cycles of denaturation at 95 °C for 2 min, annealing at 94 °C for 30 s and extension at 50 °C for 2 min. The LDR fluorescent product was analyzed using an ABI sequencer 377 (Applied Biosystems), and the results were analyzed with Genemapper software. The quality of genotyping was controlled using blinded blood duplicates.

### 3.3. Statistical Analysis

All data were statistically analyzed with the SPSS 17.0 (IBM Corporation, New York, NY, USA). Hardy-Weinberg equilibrium for each polymorphism between individual SNP was determined using the HWE program [41]. Genotype and allele frequencies of cases and controls were assessed by χ^2^-test. Linkage disequilibrium (LD) between the selected SNPs was performed using Haploview 4.2 (Broad Institute), and the frequency differences of haplotypes in patients and controls were compared using the χ^2^-test. Statistical significance was set as *p* < 0.05. Odds ratio (OR) and 95% confidence interval (CI) were calculated for the association between each genotype and AITD.

## 4. Conclusions

In brief, our study suggests that the T allele of rs7574685 and G allele of rs10181656 in STAT4 may be susceptibility factors for AITD patients in the Chinese Han population. However, the mechanisms by which two SNPs exerts their roles are not completely understood; further studies are needed to confirm or dissect the genuine functional variants of STAT4 SNPs and to understand how these polymorphisms affect AITD. More in-depth research should also be done to identify all genetic risk factors or gene-gene interactions contributing to the risk of AITD, thus, it is therefore important to continue AITD genetic studies.
